# Patient adherence with infection control measures on a novel ‘COVID-19 triage’ psychiatric in-patient ward

**DOI:** 10.1192/bjo.2021.968

**Published:** 2021-07-14

**Authors:** Ryan Williams, John Tweed, Laura Rebolledo, Osman Khalid, Josephine Agyeman, Mariana Pinto da Costa

**Affiliations:** South London and Maudsley NHS Foundation Trust, UK; and Department of Brain Sciences, Imperial College London, UK; South London and Maudsley NHS Foundation Trust, UK; South London and Maudsley NHS Foundation Trust, UK; South London and Maudsley NHS Foundation Trust, UK; South London and Maudsley NHS Foundation Trust, UK; South London and Maudsley NHS Foundation Trust, UK; and Institute of Psychiatry, Psychology and Neuroscience, King's College London, UK

**Keywords:** COVID-19, infection control, in-patient treatment, personality disorders, psychotic disorders

## Abstract

**Background:**

Intra-hospital transmission of coronavirus disease 2019 (COVID-19) is a major concern. Psychiatric in-patient units pose unique challenges for the prevention of transmission. ‘COVID-triage’ wards with strict infection control procedures have been implemented to prevent the spread of infection, but little is known about the extent to which psychiatric in-patients adhere to these procedures.

**Aims:**

To examine patient adherence with infection control measures on a novel ‘COVID-triage’ psychiatric in-patient ward in London, England.

**Method:**

This was an observational study of adherence with infection control measures. The proportion of patients who were adherent with infection control measures was calculated. The association of adherence with demographic and clinical factors was examined.

**Results:**

The majority of patients (*n* = 138/176, 78.4%) were not adherent with infection control measures. However, adherence did improve when patients who were non-adherent were given direct instructions by staff during clinical contact. Patients with diagnoses of psychotic disorders, personality disorders and substance use disorders were less likely to be adherent than those without these diagnoses.

**Conclusions:**

Psychiatric in-patients show poor adherence with infection control measures. Proactive engagement by staff is key to improving patients’ adherence. Urgent efforts are needed to identify and implement other effective methods of improving adherence in acute settings.

## Background

The ‘coronavirus disease 2019’ (COVID-19) pandemic caused by severe acute respiratory syndrome coronavirus 2 (SARS-CoV-2) is a major global public health threat, responsible for over 3.7 million deaths worldwide as of 10 June 2021.^1^

There are particular concerns regarding intra-hospital transmission as a source of infection and mortality. The majority of research to date has examined general hospitals providing acute medical and surgical care^2,3^ – but there have been no studies specifically examining the vulnerability of psychiatric in-patient facilities to viral outbreaks. The environment in many of these facilities may limit effective infection control – patients are generally confined to close-proximity living spaces where they share common dining, bathroom and recreational areas.^4^ Whereas patients in general hospitals spend the majority of time in separated bays, the nature of mental illness and models of care mean that psychiatric in-patients participate in group activities that increase patient-to-patient contact.^5^

## Concern around comorbidities

Many psychiatric patients also have poor physical health,^6–8^ with a large proportion fulfilling criteria for ‘clinical vulnerability’ to COVID-19 because of comorbidities.^9^ Disparities in mortality have been well-characterised for a range of causes for patients with severe mental illness.^10^ Of specific relevance, patients with psychotic disorders who contract influenza or pneumonia have been found to have higher mortality rates.^11^

## Intra-hospital transmission

Outbreaks of COVID-19 in psychiatric facilities in China during the emergence of the pandemic quickly highlighted the need for tailored prevention and control measures to limit intra-hospital transmission.^12,13^ Providers of mental healthcare in different countries have adopted differing strategies in order to mitigate the risks posed by COVID-19 to their psychiatric services.^14–16^ In England, some trusts (state-funded organisations providing public sector health services) have implemented a ‘COVID-triage’ system, in line with recommendations for specific ward ‘allocation and shunting’ suggested by recent studies.^17^ Under this arrangement, all new patients are admitted to a ‘COVID-triage’ ward where they are screened for COVID-19, ensuring that patients are established ‘COVID-negative’ before they can be transferred to any other in-patient bed.

This arrangement necessitates strict infection control measures on the ‘COVID-triage’ wards, to prevent patients who have been established as ‘COVID-negative’ who are awaiting transfer from being exposed to new patients who are not yet confirmed ‘COVID-negative’. Recommendations have been made for the implementation of infection control measures on psychiatric wards.^18^ However, there are concerns regarding patients’ adherence with these measures, particularly in the absence of legal frameworks to support their enforcement.^19^

## Aims

In this study we aimed to examine: (a) whether psychiatric in-patients on a novel ‘COVID-triage’ ward are adherent with infection control measures and (b) whether any patient factors were associated with poor adherence.

## The ‘COVID-triage’ ward

The ‘COVID-triage’ ward is an initiative implemented at South London and Maudsley NHS Foundation Trust (SLaM), to prevent the spread of COVID-19 in the in-patient population. SLaM is a large provider of mental healthcare covering a catchment population of around 1.2 million people in South London and provides specialist in-patient and community care for people with a wide range of mental disorders. Similar wards (albeit with different operating procedures) have been established at other psychiatric in-patient facilities in England.^20^

The ‘COVID-triage’ ward model at SLaM necessitates that all working-age patients in the Trust requiring in-patient general psychiatric treatment are admitted to one of two wards (one for men and one for women). On these wards, patients are required to either test negative for COVID-19, or complete a 14-day isolation period with no symptoms of COVID-19 (if they decline to be tested). Once established as ‘COVID-negative’ by one of these two methods, patients can then be transferred on to other wards for ongoing assessment and psychiatric treatment. This model was adapted from established ‘triage’ pathways that were originally implemented in psychiatric in-patient care to promote efficient resource utilisation.^21^

**Fig. 1:**
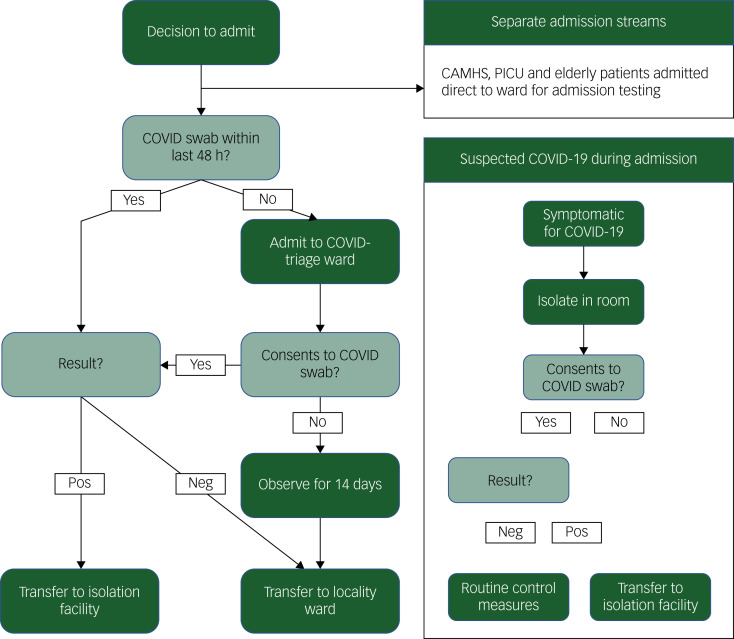
Flow chart of operational procedure for ‘COVID-triage’ model. COVID, coronavirus disease 2019; CAMHS, child and adolescent mental health services; Neg, negative; PICU, psychiatric intensive care unit; POS, positive.

Figure 1 shows a summary of the current COVID-triage arrangement (including alternative pathways for child/adolescent and older adult patients).

**Fig. 2:**
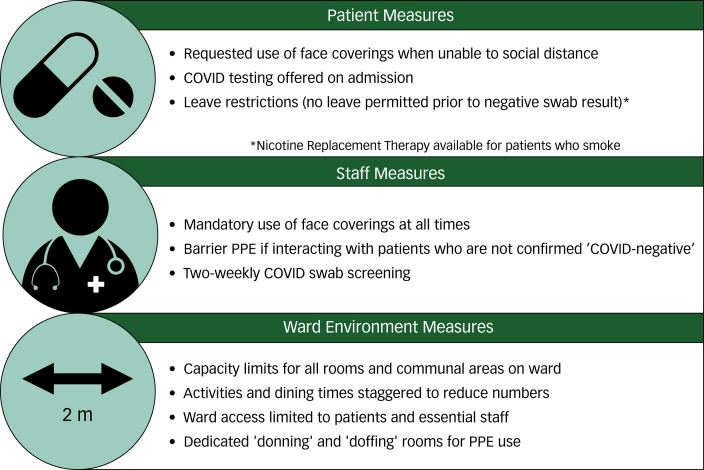
Infection control measures employed on ‘COVID-triage’ ward. COVID, coronavirus disease 2019; PPE, personal protective equipment.

During their stay on the triage ward, patients are expected to adhere to infection control measures (Fig. 2), to prevent patients who are established as ‘COVID-negative’ from significant exposure to newly arriving patients who are not yet confirmed as ‘COVID-negative’ (and therefore may be carrying SARS-CoV-2).

On arrival, during their introduction and orientation to the ward, all patients are given verbal information about the current operating procedure of the COVID-triage ward and informed of the need to comply with infection control measures during their admission. Posters are displayed on the ward to advise and remind patients and staff of the infection control measures in place. In addition, staff are advised to prompt patients (for example to don face coverings) whenever poor adherence is observed. However, these measures are not routinely enforced (for example by restraint or seclusion) for patients who refuse to comply, as such restrictive practice would not be covered by any legal framework even for patients currently detained under the Mental Health Act (2007/1983).^22^

## Method

### Study design

This was an observational cross-sectional study.

### Study approval

This study was approved by the SLaM Clinical Governance board (project reference code: 212020), who advised that it could be completed without formal ethical approval/ written consent from participants because of the project's status as an audit, and because patient identifiable data were not being recorded. The authors assert that all procedures contributing to this work comply with the ethical standards of the relevant national and institutional committees on human experimentation and with the Helsinki Declaration of 1975, as revised in 2008.

### Data collection and handling

All data for this study were gathered during an audit examining standards for infection control measures on the novel male ‘COVID-triage’ ward.

Data were collected via case-note review using the ‘electronic patient journey system’ – the digital clinical record system used by SLaM. Staff completed a password-protected data-collection tool (a spreadsheet containing variables of interest) held in a local shared drive, using information recorded in case notes.

Data were recorded for variables relating to patient demographic and clinical characteristics, as well as their adherence with infection control measures.

Data were collected during a 3-month sampling period. Data from all patients admitted during this sampling period were included in the study (i.e. no exclusion criteria) – the study therefore included data from all hospital admissions of male adults (aged 18 years and above) admitted to acute general adult services from 22 September 2020 to 21 December 2020.

### Variables of interest and covariates

The variables of interest were selected based on current national guidance for management of infection control in psychiatric in-patient units,^23^ in collaboration with members of the multidisciplinary team from the triage ward – and refined with feedback from the local Trust Clinical Governance board.

The primary variable of interest for this study was patient adherence with local infection control measures implemented on the COVID-triage ward. Patients were judged to be adherent if they maintained adequate social distancing (only possible in the ward environment by remaining in their room) or wore an appropriate ‘face covering’ when social distancing was not possible (i.e. when using common areas of the ward or clinical consulting rooms). Exceptions were made for mealtimes when face covering was not possible, but patients were expected to maintain adequate social distance.

A secondary variable of interest was whether patients were willing to don face coverings when specifically directed to do so by staff during reviews (which took place in clinical assessment rooms where social distancing was not possible).

This information was recorded for all patients during the multidisciplinary team handover meeting conducted on the ward each day.

In addition, a number of demographic and clinical variables were recorded as covariates. Respectively, these were: patient age, ethnicity, employment, accommodation status, primary diagnosis, secondary diagnosis (of personality disorder or substance use disorder), duration of primary diagnosis, whether previously admitted, whether admitted voluntarily or detained, whether ‘clinically vulnerable’ to COVID-19 (according to Public Health England definitions)^9^ and whether they were adherent with their psychiatric treatment.

### Statistical method

We used SPSS version 27^24^ to analyse the study data.

The proportion of patients who were adherent and non-adherent with infection control measures was calculated. Of these, the proportion who were willing to don face coverings when specifically asked to do so during reviews was calculated.

Descriptive statistics (mean and s.d.) were calculated for age (the only continuous variable recorded). Differences in age between patients who were and were not adherent with infection control measures were then examined using *t*-test.

Initially, the association of each covariate with adherence was examined individually using binomial logistic regression. The analysis was then repeated using multivariate logistic regression in order to adjust for potential confounding effects.

If information was missing for a variable of interest (such as employment status) the information for that variable would be coded as missing, and valid percentages are reported. These individuals were then not included in the univariate analysis pertaining to that variable. The number of missing values for each variable was very low (<5).

Covariate data were grouped where appropriate to avoid multiple small categories for the purpose of statistical analysis, i.e. primary diagnosis was recorded according to specific ICD-10 code, and grouped into psychotic versus non-psychotic disorders; ethnic categories were grouped as per the UK Census procedure.^25^

## Results

**Fig. 3:**
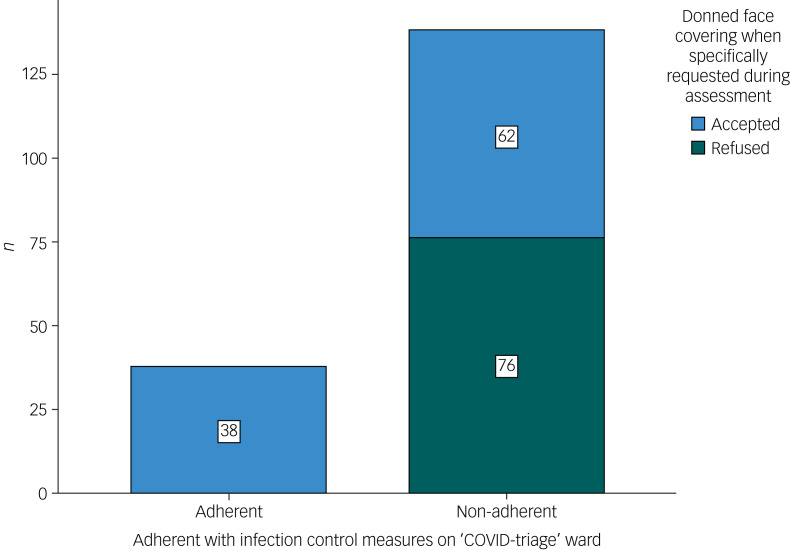
Number of patients who were adherent with infection control measures on ‘COVID-triage’ ward. COVID, coronavirus disease 2019.

Data from 176 patients’ case notes were analysed. The majority of patients (*n* = 138/176, 78.4%) were non-adherent with infection control measures. A sizeable proportion of those who were non-adherent (*n* = 62/138, 44.9%) were willing to don face coverings when specifically directed to do so by staff during reviews, although they remained non-adherent with measures during the majority of their admission (Fig. 3).

Tables 1 and 2 summarise the respective demographic and clinical characteristics of patients who were adherent with infection control measures compared with those who were non-adherent.

**Table 1:** Proportion of patients who were adherent with infection control measures – association with clinical characteristics

	*n/N* (%)	Unadjusted OR (95% CI)	*P*	Adjusted^a^ OR (95% CI)	*P*
Primary diagnosis					
Psychotic disorder	18/116 (15.5)	Ref		Ref	
Non-psychotic disorder	19/60 (31.7)	2.52 (1.20–5.29)	0.013	3.13 (1.25–7.83)	0.015
Secondary diagnosis of personality disorder					
No	35/132 (26.5)	Ref		Ref	
Yes	3/44 (6.8)	0.13 (0.03–0.57)	0.002	0.05 (0.01–0.23)	<0.001
Secondary diagnosis of substance use disorder					
No	30/100 (30.0)	Ref		Ref	
Yes	8/76 (10.5)	0.24 (0.10–0.58)	0.001	0.27 (0.11–0.70)	0.007
Duration of primary diagnosis					
>5 years	22/98 (22.4)	Ref		Ref	
<5 years	16/78 (20.5)	0.95 (0.46–1.97)	0.882	1.65 (0.69–3.99)	0.264
Previous psychiatric admissions					
Yes	23/125 (18.4)	Ref		Ref	
No	15/51 (29.4)	1.95 (0.91–4.16)	0.081	1.32 (0.54–3.24)	0.543
Admitted voluntarily or detained					
Detained	25/138 (18.1)	Ref		Ref	
Voluntary	13/38 (34.2)	2.47 (1.11–5.51)	0.024	1.52 (0.87–2.08)	0.067
Clinically vulnerable to COVID-19					
No	30/137 (21.9)	Ref		Ref	
Yes	8/39 (20.5)	0.96 (0.40–2.31)	0.929	1.4 (0.30–2.28)	0.708
Adherent with psychiatric treatment					
Yes	33/125 (26.4)	Ref		Ref	
No	5/51 (9.8)	0.4 (0.16–1.04)	0.045	0.43 (0.15–1.23)	0.114

COVID, coronavirus disease 2019; Ref, reference.

a. Adjusted for age, ethnicity, employment status, accommodation status and primary/secondary diagnosis.

**Table 2:** Proportion of patients who were adherent with infection control measures – association with demographic characteristics

	*n/N* (%)	Mean (s.d.)	Unadjusted OR (95% CI)	*P*	Adjusted^a^ OR (95% CI)	*P*
Ethnicity						
White	22/78 (28.2)	–	Ref		Ref	
Black	11/75 (14.7)	–	0.44 (0.20–0.98)	0.045	0.32 (0.13–0.82)	0.017
Mixed	1/8 (12.5)	–	0.36 (0.04–3.13)	0.357	0.15 (0.02–1.46)	0.102
Asian	2/5 (40.0)	–	1.7 (0.27–10.86)	0.577	2.48 (0.29–21.11)	0.408
Other	1/10 (10.0)	–	0.48 (0.34–2.37)	0.244	0.15 (0.02–1.42)	0.098
Employment						
Unemployed	21/101 (20.8)	–	Ref		Ref	
Long-term sick	10/50 (20.0)	–	1.01 (0.43–2.37)	0.977	1.46 (0.53–4.01)	0.466
Employed	7/25 (28.0)	–	1.58 (0.58–4.29)	0.374	1.63 (0.68–2.20)	0.457
Accommodation						
Mainstream	32/120 (26.7)	–	Ref		Ref	
Supported	5/38 (13.2)	–	0.54 (0.21–1.41)	0.207	0.89 (0.29–2.70)	0.834
Homeless	1/18 (0.1)	–	0.17 (0.12–1.32)	0.09	0.17 (0.02–1.52)	0.113
Age, years^b^		–				
Patients who were adherent	–	40.39 (14.64)	–	–	–	–
Patients who were non-adherent	–	39.05 (13.52)	–	–	–	–

COVID, coronavirus disease 2019; Ref, reference.

a. Adjusted for age, ethnicity, employment status, accommodation status and primary/secondary diagnosis.

b. Difference in age between in the two groups was not statistically significant as determined by *t*-test: *t*(174) = 0.533, *P* = 0.595.

After adjustment for covariates, patients with a recorded primary diagnosis of any non-psychotic disorder were significantly more likely to be adherent with infection control measures compared with those with diagnoses of psychotic disorders (odds ratio (OR) = 3.13, 95% CI 1.25–7.83, *P* = 0.015).

In addition, patients with a recorded secondary diagnosis of personality disorder were significantly less likely to be adherent with infection control measures compared with those without such a diagnosis (OR = 0.05, 95% CI 0.01–0.23, *P* < 0.001). Patients with a recorded secondary diagnosis of any substance use disorder were also significantly less likely to be adherent with infection control measures (OR = 0.27, 95% CI 0.11–0.70, *P* = 0.007).

Patients who were admitted to hospital involuntarily (as compared with those admitted voluntarily), patients with previous psychiatric admissions (as compared with those who had no previous admissions), patients who were non-adherent with their psychiatric treatment (as compared with those who complied with psychiatric treatment), and patients who were not classified as ‘medically vulnerable’ to severe COVID-19 infection (as compared with those who were) were less likely to be adherent with measures, but none of these differences reached statistical significance after adjustment for covariates.

From the demographic factors examined, only patient ethnicity was associated with significant differences in adherence. Patients from Black ethnic groups were specifically less likely than those from White ethnic groups to be adherent with infection control measures. Age, employment and accommodation status were not associated with significant differences in adherence. Homeless patients were very unlikely to be adherent, but when compared with those in mainstream accommodation, this difference did not reach statistical significance.

## Discussion

Patients in this ‘COVID-triage’ ward setting had poor adherence with infection control measures. However, approximately half of patients who were non-adherent were willing to abide by some measures (donning face coverings) when directly instructed to by staff, where social distancing was not possible. This indicates that relatively simple interventions appear to improve adherence for some patients.

Patients with psychotic disorders, personality disorders or substance use disorders may be less likely to comply with infection control measures.

### Strengths and limitations

The COVID-triage model is a new development in psychiatric in-patient services, and to our knowledge this is the first study describing this model of care as well as examining patients’ adherence to masks in this acute setting. Mental healthcare providers abroad have recognised the threat posed to in-patient services by intra-hospital transmission of COVID-19,^14–16^ but there is no consensus on how best to combat this.

Data were obtained from a heterogeneous sample – encompassing every male admission requiring acute general adult psychiatric in-patient care during the sampling period, at a large urban mental healthcare provider. These data therefore represent a variety of patients with different diagnoses and clinical presentations, and these results might be generalisable to similar patient groups and facilities – although possibly not to other settings where patient demographics may differ.

However, this study does have important limitations. We only gathered data from the male COVID-triage ward – so are unable to make inferences about practices on its female equivalent, or other in-patient areas that do not use a COVID-triage model (such as child and adolescent services, older adult services). Adherence was recorded as a single assessment during the handover meeting each day based on patients’ behaviour over a 24 h period. As patients spent only a short time on the COVID-triage ward before being transferred, we were unable to examine longitudinal changes in patients’ adherence during the course of their admission.

Another limitation is the relatively short period of data collection and small sample size. However, this was because of the evolving state of the pandemic and the necessity to assess and review the current procedures at organisational level. This small sample may have affected our ability to detect statistically significant differences in adherence between groups of patients where the effect size was relatively small.

In addition, data were produced from a case-note review, and were therefore dependant on accurate reporting and documentation of events at the time of occurrence – which may limit the data accuracy. Finally, as this was a cross-sectional study we are unable to fully explore the nature of associations between adherence and the other demographic and clinical variables examined. There are potential confounding variables that we were unable to examine (such as smoking status), and there may be further unidentified factors that have an impact on adherence that are responsible for the differences we observed between groups.

### Implications for clinical practice, policies and research

Interventions such as face covering and social distancing have been identified as crucial measures to reduce the nosocomial spread of COVID-19 both in the community^26^ and within hospitals.^27^ Poor adherence results in an increased potential for patients who tested negative at initial screening to contract COVID-19 during their hospital admission, and thereby spread infection to other wards when they are transferred.

There are likely to be multiple reasons for patients’ poor adherence in an acute psychiatric in-patient setting, and it is worth noting that studies of similar measures in the general population (both in the UK and abroad) have also shown variable adherence.^28,29^ Standardised guidelines are needed for the implementation of the ‘COVID-triage’ model of in-patient care, in order to safeguard patient welfare and enable staff to carry out their roles safely and effectively.^30^ These should include recommendations for infection control measures, and how best to support patients to comply with these.

We found that many patients who are generally non-adherent are willing to adopt some measures when given specific directions by staff. This indicates that changes in current clinical practice may be effective, with staff taking a more authoritative role and instructing patients for example to don face coverings). Staff training sessions may be helpful in facilitating this, by reviewing the current guidance, the importance of infection control measures and of proactively directing patients to comply with these.

However, flexible approaches that take patient factors into account may also be needed to achieve the best results. For example, in our study, patients with psychotic disorders were less likely to be adherent than those with other psychiatric disorders. This may be because of reduced awareness of current events, delusional beliefs about the intended purpose and consequences of face coverings, or retaliation against other restrictions of liberty incurred during their admission.

Our results suggest that patients with secondary diagnoses of personality disorder or substance use disorders were also less likely to be adherent with infection control measures. This may reflect existing findings that patients with these diagnoses are less likely to maintain adherence with a number of treatments.^31^ However, it may also indicate differences in the standards of care provided to these patients – particularly that staff may be less likely to engage with them or prompt them. Difficulties in establishing therapeutic alliance with both groups of patients have been well-characterised.^32–34^ Previous studies have identified variation in the standards of care provided to both groups, with particular deficits related to collaborative treatment.^35–37^

Our study found that adherence was lower among patients who were Black. This is particularly concerning in light of the established increased risk of severe disease and mortality in Black and minority ethnic group patients who contract COVID-19,^38^ and the over-representation of these groups among psychiatric in-patients.^39^ Low levels of adherence in homeless patients – although not statistically significant in itself – suggests that this vulnerable group may also require particular attention. Further investigation into adherence with infection control measures among different patient ethnic and demographic groups is required, as this may represent inadequate dissemination of public health messages (for example the current UK government initiative ‘hands face space’^40^) in particular communities.

Our findings indicate a need for further research into strategies to provide safe and effective mental health in-patient services, in the context of the ongoing pandemic. Qualitative studies to examine patients’ attitudes towards infection control measures in acute psychiatric settings would be beneficial. Studies of staff views about potential barriers to supporting patients with adherence could be equally helpful, as we found in our study that staff have a major role in patient's behaviour.

Together, these could lead to the development of targeted interventions for patients at high risk for non-adherence (i.e. patients identified as having personality disorder or substance use disorders at the point of admission). Future research could also examine patient adherence with infection control procedures in different settings (for example conventional non-triage psychiatric wards, psychiatric out-patient clinics) to explore whether patients behave differently in different contexts – and identify any reasons for this.

In conclusion, adherence with infection control measures on the novel COVID-triage ward was poor, but varied. Patients with psychotic, personality or substance use disorders were less likely to be adherent with infection control measures than those without these diagnoses. Adherence with specific measures improved when patients were instructed by staff during clinical contact. These findings merit further research into the processes underlying the observed poor adherence, and methods to improve this, in order to improve the efficacy of the COVID-triage model of care.

## Data Availability

All authors had access to the full study data-set. The data-set is held by the project supervisor and could be made available on request.

## References

[1] European Centre for Disease Prevention and Control. COVID-19 Data. ECDC, 2021 (https://www.ecdc.europa.eu/en/covid-19/data).

[2] Wang D, Hu B, Hu C, Zhu F, Liu X, Zhang J, Clinical characteristics of 138 hospitalized patients with 2019 novel coronavirus-infected Pneumonia in Wuhan, China. JAMA 2020; 323: 1061–9. 32031570 10.1001/jama.2020.1585 PMC7042881

[3] Fumagalli S, Salani B, Gabbani L, Mossello E, Ungar A. Covid-19 cases in a no-Covid-19 geriatric acute care setting. a sporadic occurrence? Eur J Intern Med 2020; 77: 141–2. 32386946 10.1016/j.ejim.2020.04.058 PMC7190479

[4] Department of Health. Health Building Guidance: Adult Acute Mental Health Units. Department of Health, 2013 (https://assets.publishing.service.gov.uk/government/uploads/system/uploads/attachment_data/file/147864/HBN_03-01_Final.pdf).

[5] Sharac J, McCrone P, Sabes-Figuera R, Csipke E, Wood A, Wykes T. Nurse and patient activities and interaction on psychiatric inpatients wards: a literature review. Int J Nurs Stud 2010; 47: 909–17. 20417514 10.1016/j.ijnurstu.2010.03.012 PMC4018996

[6] Leucht S, Burkard T, Henderson J, Maj M, Sartorius N. Physical illness and schizophrenia: a review of the literature. Acta Psychiatr Scand 2007; 116: 317–3. 17919153 10.1111/j.1600-0447.2007.01095.x

[7] McEvoy JP, Meyer JM, Goff DC, Nasrallah HA, Davis SM, Sullivan L, Prevalence of the metabolic syndrome in patients with schizophrenia: baseline results from the Clinical Antipsychotic Trials of Intervention Effectiveness (CATIE) schizophrenia trial and comparison with national estimates from NHANES III. Schizophr Res 2005; 80: 19–32. 16137860 10.1016/j.schres.2005.07.014

[8] Howell S, Yarovova E, Khwanda A, Rosen SD. Cardiovascular effects of psychotic illnesses and antipsychotic therapy. Heart 2019; 105: 1852–9. 31439658 10.1136/heartjnl-2017-312107

[9] Public Health England. Disparities in the Risk and Outcomes of COVID-19. PHE, 2020 (https://assets.publishing.service.gov.uk/government/uploads/system/uploads/attachment_data/file/908434/Disparities_in_the_risk_and_outcomes_of_COVID_August_2020_update.pdf).

[10] Thornicroft G. Premature death among people with mental illness. BMJ 2013; 346: f2969. 23674141 10.1136/bmj.f2969

[11] Olfson M, Gerhard T, Huang C, Crystal S, Stroup TS. Premature mortality among adults with schizophrenia in the United States. JAMA Psychiatry 2015; 72: 1172–81. 26509694 10.1001/jamapsychiatry.2015.1737

[12] Zhu Y, Chen L, Ji H, Xi M, Fang Y, Li Y. The risk and prevention of novel coronavirus pneumonia infections among inpatients in psychiatric hospitals. Neurosci Bull 2020; 36: 299–302. 32096116 10.1007/s12264-020-00476-9 PMC7056754

[13] Xiang YT, Zhao YJ, Liu ZH, Li XH, Zhao N, Cheung T, The COVID-19 outbreak and psychiatric hospitals in China: managing challenges through mental health service reform. Int J Biol Sci 2020; 16: 1741–4. 32226293 10.7150/ijbs.45072 PMC7098035

[14] Bojdani E, Rajagopalan A, Chen A, Gearin P, Olcott W, Shankar V, COVID-19 Pandemic: impact on psychiatric care in the United States. Psychiatry Res 2020; 289: 113069. 10.1016/j.psychres.2020.113069 PMC7200362 32413707

[15] Bureau of Disease Control and Prevention. Notice on Strengthening the Treatment and Management of Patients with Severe Mental Disorders During the Outbreak of Novel Coronavirus Pneumonia. National Health Commission of the People's Republic of China, 2020 (http://www.nhc.gov.cn/xcs/fkdt/202002/2dbbd2c1c98d4d2298bc275211b24f69.shtml).

[16] Adorjan K, Pogarell O, Streb D, Padberg F, Erdmann C, Koller G, Role of psychiatric hospitals during a pandemic: introducing the Munich Psychiatric COVID-19 Pandemic Contingency Plan. BJPsych Open 2021; 7: e41. 33517940 10.1192/bjo.2020.167 PMC7853741

[17] Hsu ST, Chou LS, Chou FHC, Hsieh KY, Chen CL, Lu WC, Challenge and strategies of infection control in psychiatric hospitals during biological disasters—from SARS to COVID-19 in Taiwan. Asian J Psychiatr 2020; 54: 102270. 32619835 10.1016/j.ajp.2020.102270 PMC7320715

[18] Barnett B, Esper F, Foster CB. Keeping the wolf at bay: infection prevention and control measures for inpatient psychiatric facilities in the time of COVID-19. Gen Hosp Psychiatry 2020; 66: 51–3. 32682153 10.1016/j.genhosppsych.2020.07.004 PMC7354767

[19] Russ MJ, Sisti D, Wilner PJ. When patients refuse COVID-19 testing, quarantine, and social distancing in inpatient psychiatry: clinical and ethical challenges. J Med Ethics 2020; 46: 579–81. 32651254 10.1136/medethics-2020-106613 PMC7371475

[20] Knowles M, Aref-Adib G, Moslehi S, Aubrey-Jones D, Obeney-Williams J, Leveson S, Containing COVID: the establishment and management of a COVID-19 ward in an adult psychiatric hospital. BJPsych Open 2020; 6: e140. 33176900 10.1192/bjo.2020.126 PMC7674787

[21] Inglis G, Baggaley M. Triage in mental health - a new model for acute in-patient psychiatry. Psychiatr Bull 2005; 29: 255–8.

[22] UK Government. Mental Health Act. UK Government, 1983. (https://www.legislation.gov.uk/ukpga/1983/20/section/50).

[23] Royal College of Psychiatrists. COVID-19: Secondary and specialist mental health settings. Royal College of Psychiatrists, 2020 (https://www.rcpsych.ac.uk/about-us/responding-to-covid-19/responding-to-covid-19-guidance-for-clinicians/community-and-inpatient-services/covid-19-working-in-secondary-and-specialist-mental-health-settings).

[24] IBM Corp. IBM SPSS Statistics for Windows, Version 27. IBM, 2020.

[25] Office for National Statistics. UK Census: List of Ethnic Groups. ONS, 2011 (https://www.ethnicity-facts-figures.service.gov.uk/style-guide/ethnic-groups).

[26] Wilder-Smith A, Freedman DO. Isolation, quarantine, social distancing and community containment: pivotal role for old-style public health measures in the novel coronavirus (2019-nCoV) outbreak. J Travel Med 2020; 27: taaa020. 32052841 10.1093/jtm/taaa020 PMC7107565

[27] Wee LE, Conceicao EP, Sim XYJ, Aung MK, Tan KY, Wong HM, Minimizing intra-hospital transmission of COVID-19: the role of social distancing. J Hosp Infect 2020; 105: 113–5. 32294511 10.1016/j.jhin.2020.04.016 PMC7152925

[28] Norman P, Wilding S, Conner M. Reasoned action approach and compliance with recommended behaviours to prevent the transmission of the SARS-CoV-2 virus in the UK. Br J Health Psychol 2020; 25: 1006–19. 33007143 10.1111/bjhp.12474 PMC7536976

[29] Clark C, Davila A, Regis M, Kraus S. Predictors of COVID-19 voluntary compliance behaviors: an international investigation. Glob Transitions 2020; 2: 76–82. 10.1016/j.glt.2020.06.003 PMC7318969 32835202

[30] Gan WH, Lim JW, Koh D. Preventing intra-hospital infection and transmission of coronavirus disease 2019 in health-care workers. Saf Health Work 2020; 11: 241–3. 32292622 10.1016/j.shaw.2020.03.001 PMC7102575

[31] Pompili M, Venturini P, Palermo M, Stefani H, Seretti ME, Lamis DA, Mood disorders medications: predictors of nonadherence – review of the current literature. Expert Rev Neurother 2013; 13: 809–25. 23898852 10.1586/14737175.2013.811976

[32] Palmer RS, Murphy MK, Piselli A, Ball SA. Substance user treatment dropout from client and clinician perspectives: a pilot study. Subst Use Misuse 2009; 44: 1021–38. 19938942 10.1080/10826080802495237 PMC3678276

[33] Fanaian M, Lewis KL, Grenyer BFS. Improving services for people with personality disorders: views of experienced clinicians. Int J Ment Health Nurs 2013; 22: 465–71. 23294488 10.1111/inm.12009

[34] Woollaston K, Hixenbaugh P. ‘Destructive whirlwind’: nurses’ perceptions of patients diagnosed with borderline personality disorder. J Psychiatr Ment Health Nurs 2008; 15: 703–9. 18844794 10.1111/j.1365-2850.2008.01275.x

[35] Watkins KE, Burnam A, Kung F-Y, Paddock S. A national survey of care for persons with co-occurring mental and substance use disorders. Psychiatr Serv 2001; 52: 1062–8. 11474052 10.1176/appi.ps.52.8.1062

[36] Williams R, Farquharson L, Rhodes E, Dang M, Butler J, Quirk A, Impact of substance use disorder on quality of inpatient mental health services for people with anxiety and depression. J Dual Diagn 2021; 17: 80–93. 33048661 10.1080/15504263.2020.1825892

[37] Williams R, Farquharson L, Rhodes E, Dang M, Fitzpatrick N, Quirk A, Impact of co-morbid personality disorder on quality of inpatient mental health services for people with anxiety and depression. Personal Ment Health 2020; 14: 336–49. 32424943 10.1002/pmh.1484

[38] Aldridge RW, Lewer D, Katikireddi SV, Mathur R, Pathak N, Burns R, Black, Asian and Minority Ethnic groups in England are at increased risk of death from COVID-19: indirect standardisation of NHS mortality data. Wellcome Open Res 2020; 5: 88. 32613083 10.12688/wellcomeopenres.15922.1 PMC7317462

[39] Koffman J, Fulop NJ, Pashley D, Coleman K. Ethnicity and use of acute psychiatric beds: one-day survey in North and South Thames regions. Br J Psychiatry 1997; 171: 238–41. 9337976 10.1192/bjp.171.3.238

[40] Public Health England. Hands. Face. Space. - Coronavirus Resource Centre. PHE, 2020 (https://coronavirusresources.phe.gov.uk/Hands-Face-Space-/).

